# An Investigation of Vehicle Behavior Prediction Using a Vector Power Representation to Encode Spatial Positions of Multiple Objects and Neural Networks

**DOI:** 10.3389/fnbot.2019.00084

**Published:** 2019-10-16

**Authors:** Florian Mirus, Peter Blouw, Terrence C. Stewart, Jörg Conradt

**Affiliations:** ^1^BMW Group, Research, New Technologies, Garching, Germany; ^2^Department of Electrical and Computer Engineering, Technical University of Munich, Munich, Germany; ^3^Applied Brain Research Inc., Waterloo, ON, Canada; ^4^Department of Computational Science and Technology, KTH Royal Institute of Technology, Stockholm, Sweden

**Keywords:** vehicle prediction, long short-term memories, artificial neural networks, vector symbolic architectures, online learning, spiking neural networks

## Abstract

Predicting future behavior and positions of other traffic participants from observations is a key problem that needs to be solved by human drivers and automated vehicles alike to safely navigate their environment and to reach their desired goal. In this paper, we expand on previous work on an automotive environment model based on vector symbolic architectures (VSAs). We investigate a vector-representation to encapsulate spatial information of multiple objects based on a convolutive power encoding. Assuming that future positions of vehicles are influenced not only by their own past positions and dynamics (e.g., velocity and acceleration) but also by the behavior of the other traffic participants in the vehicle's surroundings, our motivation is 3-fold: we hypothesize that our structured vector-representation will be able to capture these relations and mutual influence between multiple traffic participants. Furthermore, the dimension of the encoding vectors remains fixed while being independent of the number of other vehicles encoded in addition to the target vehicle. Finally, a VSA-based encoding allows us to combine symbol-like processing with the advantages of neural network learning. In this work, we use our vector representation as input for a long short-term memory (LSTM) network for sequence to sequence prediction of vehicle positions. In an extensive evaluation, we compare this approach to other LSTM-based benchmark systems using alternative data encoding schemes, simple feed-forward neural networks as well as a simple linear prediction model for reference. We analyze advantages and drawbacks of the presented methods and identify specific driving situations where our approach performs best. We use characteristics specifying such situations as a foundation for an online-learning mixture-of-experts prototype, which chooses at run time between several available predictors depending on the current driving situation to achieve the best possible forecast.

## 1. Introduction

The race to autonomous driving is currently one of the main forces for pushing research forward in the automotive domain. With highly automated vehicle prototypes gradually making their way to our public roads and fully-automated driving on the horizon, it seems to be a matter of time until we see robot taxis or cars navigating us through urban traffic or heavy stop-and-go on highways. One major reason for this development in recent years is the rapid progress of artificial intelligence (AI), especially the success of deep learning, which has shown remarkable results in tasks essential for automated driving like object detection, classification (Ciresan et al., 2012) and control (Bojarski et al., 2016).

Predicting future behavior and positions of other traffic participants from observations is essential for collision avoidance and thus safe motion planning, and needs to be solved by human drivers and automated vehicles alike to reach their desired goal. However, future positions of vehicles not only depend on each vehicle's own past positions and dynamics like velocity and acceleration, but also on the behavior of the other traffic participants in the vehicle's surroundings. Motion prediction for intelligent vehicles in general has seen extensive research in recent years (Polychronopoulos et al., 2007; Lawitzky et al., 2013; Lefèvre et al., 2014; Schmüdderich et al., 2015) as it is a cornerstone for collision-free automated driving. Lefèvre et al. (2014) classify such prediction approaches into three categories, namely *physics-based, maneuver-based*, and *interaction-aware*, depending on their level of abstraction. *Physics-based* and *maneuver-based* motion models consider the law of physics and the intended driving maneuver, respectively as the only influencing factors for future vehicle motion and ignore inter-dependencies between the motion of different vehicles. There exist a growing number of different *interaction-aware* approaches to account for those dependencies and mutual influences between traffic participants or, more generally, agents in the scene. Probabilistic models like costmaps (Bahram et al., 2016) account for physical constraints on the movements of the other vehicles. Classification approaches categorize and represent scenes in a hierarchy (Bonnin et al., 2012) based on the most generic ones to predict behavior for a variety of different situations.

Data-driven approaches to behavior prediction mainly rely on long short-term memory (LSTM) neural network architectures (Hochreiter and Schmidhuber, 1997), which have proven to be a powerful tool for sequential data analysis. Alahi et al. (2016) model interactions in the learning network architecture by introducing so-called social-pooling layers to connect several LSTM each predicting the distribution of the trajectory position of one agent at a time. Deo and Trivedi (2018a) adapted the combination of LSTM networks for encoding vehicle trajectories and (convolutional) social-pooling layers to account for interactions to vehicle prediction in highway situations. Altche and de La Fortelle (2017) use a LSTM network as well, but they account for interaction by including distances between the target vehicle and other agents directly in the training data rather than adapting the network architecture. A similar approach is proposed by Deo and Trivedi (2018b), but they combine LSTM networks with an additional maneuver classification network to predict future vehicle motion. One issue in data-driven approaches to behavior prediction accounting for relations between agents is that the number of other vehicles is variable. If information about vehicles other than the target are encapsulated in the input of the neural network, typically a specific number of other vehicles of interest are manually chosen a priori to avoid this issue (Altche and de La Fortelle, 2017; Deo and Trivedi, 2018b). If the information about other vehicles is encapsulated in the network architecture, it might be necessary to train several networks depending on the situation at hand.

**Figure 1:**
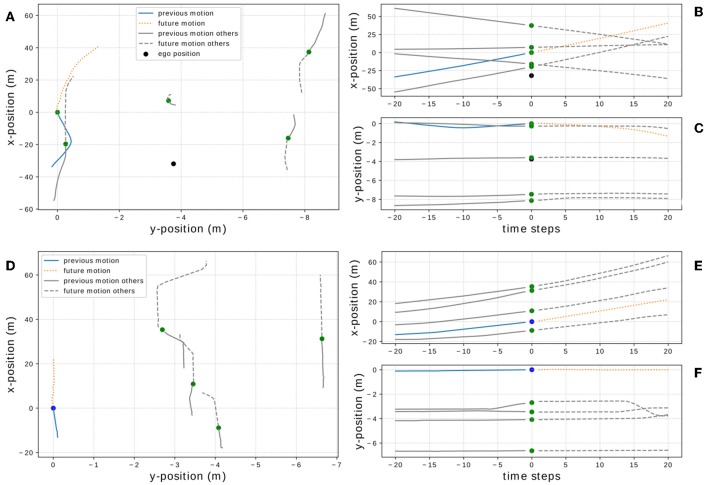
Data visualization of one driving situation example from the *On-board* data set *D*_1_ **(A–C)** and one example from the *NGSIM* data set *D*_2_ **(D–F)**. The dots indicate the position of the vehicles and color-code the vehicle type (red = motorcycle, green = car, blue = truck, black = ego-vehicle), blue and orange lines show past and future motion of the target vehicle whereas gray lines depict the other vehicles' motion.

In this paper, we expand our previous work (Mirus et al., 2018) on an automotive environment model based on VSAs (Gayler, 2003). In contrast to the representation shown in Mirus et al. (2018), this paper introduces a more sophisticated way of encapsulating spatial information of multiple objects in semantic vectors of fixed length. Therefore, we employ generalized exponentiation of high-dimensional vectors, referred to as the convolutive power, based on the VSA's binding operation, which in our case is circular convolution. We hypothesize that structured vector representations will be able to capture relations and mutual influence between traffic participants. For instance, in a situation as shown in Figures 1A–C, the behavior of the target vehicle, as depicted by a solid blue and dotted orange line for past and future positions, respectively, is influenced by the surrounding vehicles, as illustrated by solid and dotted gray lines for past and future positions, respectively. The target vehicle is approached from behind by a faster vehicle causing it to eventually change its lane to the right, which, for now, is still occupied by the ego-vehicle and another vehicle. All of these external influences have an impact on the target vehicle's motion (and vice versa). As we aim for a model-free data representation, we avoid introducing any physical constraints or restrictions regarding our data-representation or the predicting models.

In this work, we consider our main contributions to be the following: we present a representation of spatial information for multiple objects in semantic vectors of fixed length using the convolutive power. We use this representation as input for simple feed-forward neural networks and more sophisticated LSTM-based models and compare them against each other and a linear predictor as the simplest baseline. We conduct a thorough and detailed analysis for all of these models and show that by using our vector representation with a simple network architecture we can achieve results comparable to more complex models. This is particularly interesting for mobile applications, such as automated driving: combining our vector representation, which allows to encode spatial positions of several objects as well as efficient implementation in spiking neural networks (SNNs) (Eliasmith, 2013), with a simple feed-forward SNN would allow future deployment on dedicated, energy-efficient neuromorphic hardware. In case the performance of the simpler feed-forward networks is close enough to the more sophisticated ones, the possibility of efficient deployment could be an advantage over LSTM networks, which are by design harder to apply to dedicated computing hardware (Chang and Culurciello, 2017). Finally, we present a prototype of a mixture-of-experts online learning system, that chooses at run-time between several models, which have been pre-trained offline, to achieve the best possible forecast. We show, that this online learning approach is able to improve its performance compared to the individual prediction models.

## 2. Materials and Methods

### 2.1. Vector Symbolic Architectures

The term vector symbolic architectures (VSAs)—first coined by Gayler (2003)—refers to a family of approaches for cognitive modeling making use of distributed representations. The basic idea behind all of those approaches is to represent structure (e.g., cognitive concepts, symbols, or language) in a high-dimensional vector space by mapping each entity to be represented to a (possibly random) vector. One strength of VSAs is that they offer the possibility to manipulate their entities through algebraic operations, typically one *addition-like* and *multiplication-like* operation each. Vectors, which represent basic concepts not intended to be further decomposable and thus are not constructed from other vectors using the VSA's algebraic operations, are called *atomic vectors*.

#### 2.1.1. Prerequisites

In this paper, we adopt the semantic pointer architecture (SPA) (Eliasmith, 2013), a variant of holographic reduced representations (HRRs) originally introduced by Plate (1994), to encode automotive scenes in high-dimensional vectors (note: the source-code for all models presented in this paper can be found at https://github.com/fmirus/spa_trajectory_prediction). Thus, atomic vectors are picked from the real-valued unit sphere and the dot product serves as a measure of similarity. We call two vectors *similar*, if their dot-product is higher than a certain similarity threshold. The distribution of the dot-product of two randomly chosen unit vectors has a mean of 0 and a standard deviation of $\frac{1}{\sqrt{D}}$ (Widdows and Cohen, 2014). Thus, the similarity threshold is typically chosen as $\frac{c}{\sqrt{D}}$ for some constant *c*, which is a similarity value that we would expect from two randomly chosen vectors and only depends on the dimension *D* of the vector space. Furthermore, the algebraic operations are component-wise vector addition ⊕ and circular convolution ⊛, which is defined as

(1) $$\begin{array}{r} \text{z}=\text{v}⊛\text{w}\text{ with}z_{j}:=\overset{D-1}{\underset{k=0}{\sum }}v_{k}w_{\left(j-k\right)\text{ mod }D} \end{array}$$

for any two *D*-dimensional vectors **v**, **w**. One important property of this operation is the fact (Bracewell, 2000, Chapter 6), that circular convolution can efficiently be computed using the discrete Fourier transform:

(2) $$\begin{array}{r} \text{v}⊛\text{w}=IDFT\left(DFT\left(\text{v}\right)⊙DFT\left(\text{w}\right)\right), \end{array}$$

where ⊙ denotes element-wise multiplication, DFT and IDFT denote the discrete Fourier transform and inverse discrete Fourier transform, respectively. The neutral element regarding circular convolution is **1** = (1, 0, ⋯ , 0). Furthermore, for any vector **v**, the vector $\bar{\text{v}}=\left(v_{0},v_{D-1},\ldots ,v_{1}\right)$ is a *pseudo-inverse* element with respect to circular convolution, meaning that the vector derived from convolving them is similar to the neural element, i.e., $\text{v}⊛\bar{\text{v}}\approx \text{1}$. Although we can also find an exact inverse element **v**^−1^ for most vectors with **v** ⊛ **v**^−1^ = **1**, it is often more useful to work with pseudo-inverses instead of exact inverse elements, as they can become unstable in certain situations. However, we call the special class of vectors for which the pseudo- and exact inverse element coincide *unitary vectors*, i.e., ${\text{v}}^{-1}=\bar{\text{v}}$.

Using Equation (2), we define the *convolutive power* of a vector *v* by an exponent *p* ∈ ℝ as

(3) $$\begin{array}{r} {\text{v}}^{p}:=ℜ\left(IDFT\left(({DFT}_{0}{\left(\text{v}\right)}^{p}),\ldots ,({DFT}_{D-1}{\left(\text{v}\right)}^{p}\right)\right)), \end{array}$$

where ℜ denotes the real part of a complex number and *DFT*_*i*_ (**v**) denotes the *i*th component of the vector *DFT* (**v**). Denoting the set of unitary vectors by U, we state three essential properties

- All elements of U have unit length, i.e., we have ||**u**|| = 1 for any vector **u** ∈ U.
- U is closed under convolutive exponentiation, i.e., **u**^*p*^ ∈ U for any **u** ∈ U and *p* ∈ ℝ.
- Convolution with unitary vectors preserves the norm, i.e., ||**v**|| = ||**v** ⊛ **u**|| for any **v** and any unitary vector **u** ∈ U.

#### 2.1.2. Convolutive-Power Representation

In this paper, we adopt and improve the vector representation for automotive scenes introduced in earlier work (Mirus et al., 2018). Here, we introduce the convolutive vector-power shown in Equation (3) for encoding spatial positions of multiple vehicles and focus on investigating its expressive power. To create a vocabulary *V* of atomic vectors, we assign a random real-valued vector from the unit sphere to each category of dynamic objects (e.g., car, motorcycle, truck) as well as random unitary vectors **X** and **Y** to encode spatial positions. We use unitary vectors for **X** and **Y** as they have unit length and are closed under convolutive exponentiation. Therefore, by encoding spatial positions with powers of unitary vectors, we avoid exploding lengths of our final scene vectors, which would lead to additional noise and unwanted behavior when using them as input for neural networks. Furthermore, we use additional random ID-vectors **TARGET** and **EGO** representing the target object to be predicted and, if applicable, the ego-vehicle, respectively.

**Figure 2:**
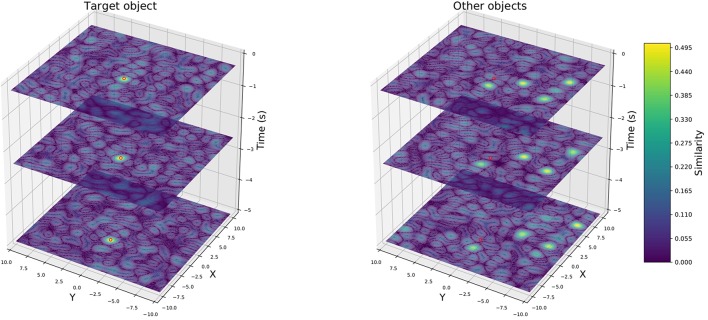
Visualization of the convolutive vector-power representation of one particular driving situation over time at selected time-steps as a heat map of similarity values for 512-dimensional vectors. The red circles indicate the measured position of the target vehicle.

Given a situation as shown in Figure 1A with a sequence of prior positions (*x*_*t*_, *y*_*t*_) for the target vehicle at time step *t* ∈ {*t*_0_, …, *t*_*N*_} and equivalent sequences (*x*_*obj,t*_, *y*_*obj,t*_) for other traffic participants, we encapsulate this positional information in a scene vector

(4) $$\begin{array}{r} {\text{S}}_{t}=\underset{\text{target-vehicle}}{\underset{︸}{\text{TARGET}⊛{\text{TYPE}}_{target}⊛{\text{X}}^{x_{t}}⊛{\text{Y}}^{y_{t}}}}\\ \;⊕\underset{\text{other objects}}{\underset{︸}{\underset{obj}{\sum }{\text{TYPE}}_{obj}⊛{\text{X}}^{x_{obj,t}}⊛{\text{Y}}^{y_{obj,t}}}} \end{array}$$

for each time step *t*. This yields a sequence of semantic scene vectors **S**_*t*_ for *t* ∈ {*t*_0_, …, *t*_*N*_} encoding the past spatial development of objects in the current driving situation. Figure 2 depicts the aforementioned scene vector representation: the left plots show similarities (depicted as heat map) between the vector **S**_*t*_ encoding the scene from Figure 1A and the vectors $v_{i}=\text{TARGET}⊛{\text{TYPE}}_{target}⊛{\text{X}}^{{\bar{x}}_{i}}⊛{\text{Y}}^{{ȳ}_{i}}$ for a sequence of discrete position samples ${\bar{x}}_{i},{ȳ}_{i}$. Similarly, the right plots show similarities between **S**_*t*_ and $\text{CAR}⊛{\text{X}}^{{\bar{x}}_{i}}⊛{\text{Y}}^{{ȳ}_{i}}$ visualizing all other objects in the scene of type *car*. We observe clear peaks (bright yellow areas) of higher similarities at the true positions of the encoded objects depending on their type (e.g., car or truck) or if the object is the target object of interest. Hence, we can encode spatial information of several different objects in a sequence of semantic vectors and reliably decode it back out. This allows us to encode automotive scenes with varying number of dynamic objects in a vector representation of fixed dimensionality.

### 2.2. Models

#### 2.2.1. LSTM Networks

**Figure 3:**
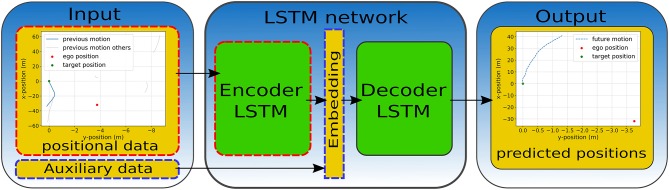
Visualization of our LSTM-based learning architecture. Modules that change with varying encoding scheme of the input data are highlighted through dashed red borders whereas parts that change when varying the data set are highlighted through dashed blue borders.

In this work, we use a long short-term memory (LSTM) (Hochreiter and Schmidhuber, 1997) network-architecture for the prediction of vehicle positions. Our network consists of one LSTM encoder and decoder cell for sequence to sequence prediction, which means that the input and the final result of our model is sequential data. The encoder LSTM takes positional data for 20 past, equidistant time frames as input. That is, the input data is a sequence of 20 items of either positions of the target vehicle or a sequence of high-dimensional vectors encoding this positional data (see sections 1, 2.3.4 for further details). Thus, the resulting embedding vector encodes the history of the input data over those 20 time frames. This embedding vector is concatenated with additional auxiliary information to aid the model when predicting the future trajectory of the target vehicle. This auxiliary data is information, that is available to the system when the prediction is to happen, i.e., sensory data available at prediction time or future data about the ego-vehicle, such as its own planned trajectory (see section 3.1.1 for further details on this auxiliary data). Finally, the embedding vector is used as input for the decoder LSTM to predict future vehicle positions. The output of each model is a sequence of 20 positions of the target vehicle predicted over a certain temporal horizon into the future. We use the same network architecture for all encoding schemes of the input data and for both data sets. However, the dimensionality of the input varies over the different encoding schemes while the auxiliary information used to enrich the embedding vector is different depending on the data set (since only one data set is recorded from a driving ego-vehicle). We describe these implementation choices in more detail in section 3.1.1. Figure 3 visualizes the architecture of our LSTM models indicating modules that change when varying the encoding scheme by a dashed red border whereas parts that change with the data set are highlighted through a dashed blue border.

#### 2.2.2. NEF Networks

As an alternative to the LSTM-models, we also considered a much simpler single-hidden-layer network defined using the neural engineering framework (NEF) (Eliasmith and Anderson, 2003). While this is usually used for constructing large-scale biologically realistic neuron models (Eliasmith et al., 2012), the NEF software toolkit Nengo (Bekolay et al., 2014) also allows for traditional feed-forward artificial neural networks using either spiking or non-spiking neurons. Spiking neurons are of considerable interest for vehicle prediction algorithms due to the potential for reduced power consumption when run on hardware that is optimized for spiking neurons (i.e., neuromorphic hardware).

For these NEF networks, we use a single hidden layer containing *N* neurons, with randomly generated (and fixed) input weights, and use least-squares optimization to compute the output weights. That is, given the hidden layer spiking activity *a*_*i*_ for the *i*th neuron (i.e., a sequence of spikes)

(5) $$\begin{array}{r} a_{i}\left(\text{x}\left(t\right)\right)=\overset{m_{i}}{\underset{j=1}{\sum }}h\left(t\right)*\delta \left(t-t_{j}\right)=\overset{m_{i}}{\underset{j=1}{\sum }}h\left(t-t_{j}\right), \end{array}$$

where δ denotes the delta function, *h*(*t*) is the post-synaptic current produced by a single spike and *t*_*j*_ are the *m*_*i*_ spike times of the *i*th neuron, we compute the network output **y** with output weights **d**_*i*_ as

(6) $$\begin{array}{r} \text{y(t)}=\overset{N}{\underset{i=1}{\sum }}a_{i}\left(\text{x}\left(t\right)\right){\text{d}}_{i}. \end{array}$$

If we have a desired **y**(*t*) for every given input to the network, then we can provide that input, measure the resulting hidden layer activity for each input, and then find the optimal **d**_*i*_ values to make the network output match the desired output. This is a much faster alternative to using gradient descent rules (such as backpropagation). In particular, we find the **d**_*i*_ that minimize

(7) $$\begin{array}{r} E=\int {\left(\text{y}\left(t\right)-\overset{N}{\underset{i=1}{\sum }}a_{i}\left(\text{x}\left(t\right)\right){\text{d}}_{i}\right)}^{2}\text{d}\text{x}\left(t\right). \end{array}$$

As with any traditional network, we can have any number of input, output, and hidden neurons, all following this same process. The goal here is to provide a simple baseline for comparison to the LSTM networks, to see what (if any) performance gain is produced by the more complex network approach.

#### 2.2.3. Mixture-of-Experts Online Learning

Given that we have multiple models *p*_*i*_ for *i* = 1, …, *M* for predicting vehicle positions, we also define mixture-of-experts models. These are models where the output is a weighted sum of the outputs from other models

(8) $$\begin{array}{r} {\text{v}}_{mix,t}=\underset{p}{\sum }{\text{W}}_{p,t}{\text{v}}_{p,t}, \end{array}$$

where **W**_*p,t*_ is the weight and **v**_*p,t*_ is the output value of the prediction model *p* for prediction time *t*. If each model produces a prediction of the *x* and *y* positions at *N* different time steps into the future and we have *M* models, *w* will be an *M* × *N* × 2 tensor. In other words, the particular weighting of models for predicting 0.5 s into the future may be very different from the weighting when predicting 5.0 s into the future.

The simplest way to generate these weights is to use standard delta-rule learning

(9) $$\begin{array}{r} \Delta {\text{W}}_{p,t}=\kappa {\text{v}}_{p,t}\underset{=\epsilon_{t}}{\underset{︸}{\left({\text{v}}_{observed,t}-{\text{v}}_{mix,t}\right)}}=\kappa {\text{v}}_{p,t}\epsilon_{t}. \end{array}$$

where κ is a learning rate and ϵ_*t*_ = **v**_*observed,t*_−**v**_*mix,t*_ is the current prediction error, that is, the error between the mixture model's output **v**_*mix,t*_ and the target vehicle's actual position **v**_*observed,t*_. For this paper, we initialize the weights **W**_*p,t*_ to be 1/*M* (i.e., an equal weighting across all *M* models).

The above model attempts to find the best weighting of the offline models based only on the prediction error. However, it is also possible to learn a weighting that is based on the current *context*. That is, instead of learning **W**, we can learn the function *f*_**W**_(**c**) = **W**, where **c** is some currently available sensor information.

Since neural networks are good function approximators, we implement this context-sensitive mixture-of-experts model as a single-hidden-layer artificial neural network (ANN) whose inputs are **c** and whose outputs are **W**. As with the context-free mixture-of-experts model, we initialize the output to always produce 1/*M*, and then train the network based on the prediction error.

Importantly, this context-sensitive mixture-of-experts model is meant to be trained *online*. That is, we do not pre-train it on a large corpus of data and then fix the final weights. Instead, we run the neural network training *while the system is running*, just like the context-free version. Indeed, the context-free version is equivalent to the context-sensitive model if the context is kept constant.

While any neural network learning algorithm could be used here, for simplicity we use delta rule again, and note that the delta rule is the first step of the classic backpropagation neural network learning algorithm. In other words, we only adjust the weights between the hidden layer and the output layer, and leave the other set of weights at their initial randomly generated values. This greatly reduces the computation cost of performing the online learning.

**Figure 4:**
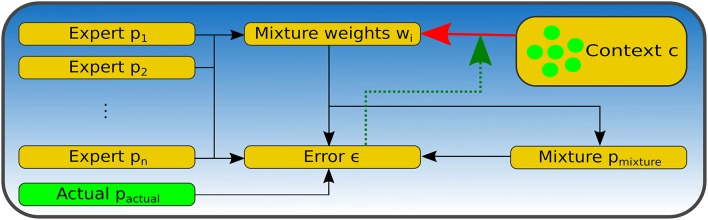
Visualization of the network architecture of the context-sensitive mixture-of-experts online learning system. Yellow boxes indicate the individual components of the model, while the solid red line depicts the connection to decode out the weights **W**_*p,t*_ for the individual expert predictors from the neural population encoding the context **c** as indicated by the green circles in the context component. The dotted green arrow indicates that the error signal is used to update the weights of this connection using delta-rule learning.

Figure 4 shows a schematic visualization of the mixture-of-expert model's architecture. Yellow boxes indicate the individual components of the model, while the solid red line depicts the connection to decode out the weights **W**_*p,t*_ for the individual expert predictors from the neural population encoding the context **c** as indicated by the green circles in the context component. Finally, the dotted green arrow indicates that the error signal is used to update the weights of this connection using delta-rule learning.

### 2.3. Data and Pre-processing

In this work, we use two different data sets for training and evaluation of our system, which we describe in more detail in the subsequent sections. We refer to those data sets as *On-board* or *D*_1_ (see section 2.3.1), which is our main data set, and *NGSIM* or *D*_2_ (see section 2.3.2), which is a publicly available data set used for reference and comparability. In this section, we describe both data sets regarding available features, available sources of information as well as their key differences and the preprocessing procedure.

#### 2.3.1. On-Board-Sensors Data Set

This is our main data set used in this work. It contains real-world data gathered using the (ego-) vehicle's on-board sensors during test drives mainly on highways in southern Germany. The data contains object-lists with a variety of features obtained from different sensor sources. Apart from features about motion and behavior of the dynamic objects in the scene like position, velocity and acceleration, which are estimated from light detection and ranging (LIDAR) sensors, there is also visual information like object type probabilities or lane information, which is acquired from additional camera sensors. More detailed information on the test vehicle's sensor setup can be found in Aeberhard et al. (2015). The fused information about objects is available at a frequency of roughly 5 Hz. The main feature of this data set is that all information about other vehicles, such as position or velocity are measured with respect to the ego-vehicle and its coordinate system. The *On-board* data set contains 3,891 vehicles, which yield a total length of roughly 28.3 h when adding up the time each individual vehicle is visible.

#### 2.3.2. NGSIM US-101 Data Set

The next generation simulation (NGSIM) US-101 data set (Colyar and Halkias, 2017) is a publicly available data set recorded on a segment of ~640 m length with 6 lanes on the US-101 freeway in Los Angeles, California. Although the data set was originally intended for driver behavior and traffic flow models (He, 2017), it has also been used to train trajectory predictions models (Altche and de La Fortelle, 2017; Deo and Trivedi, 2018b). The data set was recorded using cameras observing freeway traffic from rooftops with trajectory-data being extracted later from the obtained video footage. It holds a total of 45 min of driving data split into three 15 min segments of mild, moderate and congesting traffic conditions. Apart from positional information in lateral and longitudinal direction (in a global and local coordinate system), additional features like instantaneous velocity, acceleration, vehicle size as well as the current lane are available for each vehicle. The trajectory data is sampled with a frequency of 10 Hz. The main difference to the *On-board* data set is the fact, that the *NGSIM* data set is recorded with an external stationary camera instead of on-board sensors of a driving vehicle. Thus, there is no ego-vehicle present in the data and all information are available in absolute coordinates instead of being measured relative to one particular ego-vehicle. The *NGSIM* data set contains 5,930 vehicles and therefore a total time of roughly 91.3 h when adding up the time each individual vehicle is visible.

#### 2.3.3. Pre-processing

In this section, we describe the preprocessing steps performed a priori to prepare the information from our two data sets as neural network input. Although we aim to keep these preprocessing steps as consistent as possible across the data sets, there are some mild differences, which we will also point out. We aim to predict future positions of dynamic objects 5 s into the future based on their positions 5 s prior to their current location. As the two data sets are sampled at different frequencies, we interpolate the available data over 20 equidistant steps to achieve intervals of 0.25 s to improve consistency and comparability. Furthermore, we translate the current position of the target vehicle (the vehicle to be predicted) into the origin, i.e., position (0, 0) (see Figure 1), to prevent our models from treating similar trajectories differently due to positional variations. Finally, to improve suitability of the data as input for neural networks, we divide all *x*-positions by a factor of 10 such that *x*-/*y*-values are scaled to a similar order of magnitude. Thus, one data sample consists of a sequence of length 20 of positional information over the past 5 s, which is used as input for our models with different encoding, and a sequence of 20 positions 5 s into the future used as labels or ground truth for the models to be trained with. For the *NGSIM* data set *D*_2_, we use only every 10th data point, to avoid the creation of too many overlapping, and therefore too similar, data samples. Furthermore, we converted all values to the metric system and swapped the dimensions of the positions in *D*_2_ such that for both data sets *x*- and *y*-direction correspond to longitudinal and lateral positions, respectively. For training and evaluating our models, we split both data sets into training *T*_*i*_ ⊂ *D*_*i*_ and validation data *V*_*i*_ ⊂ *D*_*i*_ containing 90% and 10% of the objects, respectively to avoid testing our models on vehicles they have been trained with.

#### 2.3.4. Encoding Schemes

We use different encoding schemes of the positional input data in this work. The main encoding scheme is the convolutive vector-power representation as depicted in section 2.1.2. To avoid accumulation of noise while focusing on the vehicles most relevant for prediction, we only use objects closer than 40 m to the target vehicle in the *On-board* data set. For the *NGSIM* data set *D*_2_, we additionally include only objects on the same lane as the target vehicle and on adjacent lanes. Thereby, we aim for consistency across both data sets and we keep the input data as comparable as possible to what a driving vehicle could be able to detect using its on-board sensors.

For the *On-board* data set *D*_1_, we use two different variants of this representation, which differ in that the ego-vehicle's position is used or excluded in the *other objects* part of Equation (4), yielding two sequences ${\left({\text{S}}_{t}^{ego}\right)}_{t_{0}}^{t_{N}}$ and ${\left({\text{S}}_{t}\right)}_{t_{0}}^{t_{N}}$. We used Nengo's SPA package for implementation and therefore refer to these encoding two schemes ${\left({\text{S}}_{t}\right)}_{t_{0}}^{t_{N}}$ and ${\left({\text{S}}_{t}^{ego}\right)}_{t_{0}}^{t_{N}}$ as “SPA-power” and “SPA-power-with-ego,” respectively. As the *NGSIM* data set *D*_2_ does not contain an ego-vehicle, we only investigate the “SPA-power” encoding scheme there.

For a simple reference vector-representation, we add the positional vectors **X** and **Y** scaled with the target vehicle's prior positions (*x*_*t*_, *y*_*t*_) at each time step *t*, yielding the sequence ${\tilde{\text{S}}}_{t}=x_{t}\cdot \text{X}+y_{t}\cdot \text{Y}$. Finally, we also use plain numerical position values *p*_*t*_ = (*x*_*t*_, *y*_*t*_) as input data. Note, that only the SPA-power representation variants ${\left({\text{S}}_{t}\right)}_{t_{0}}^{t_{N}}$ and ${\left({\text{S}}_{t}^{ego}\right)}_{t_{0}}^{t_{N}}$ contain positional information about vehicles other than the target.

## 3. Experiments and Results

**Figure 5:**
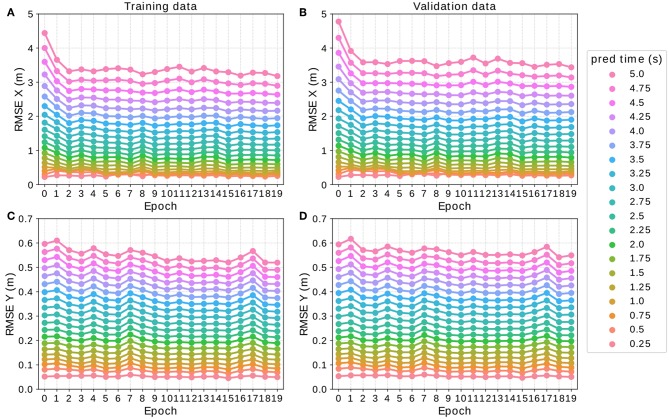
Development of the RMSE at every prediction time step during the training process of the LSTM model using the SPA-power-with-ego vector representation (LSTM SPA 3) after each epoch on the training **(A,C)** and validation part **(B,D)** of the *On-board* data set. Each colored line illustrates the RMSE of the model for one particular prediction time step while all points with the same value on the *x*-axis depict the model's performance after the respective epoch during the training process. One observes comparable trends on both training and validation set and that the RMSE stagnates after 10 epochs.

**Figure 6:**
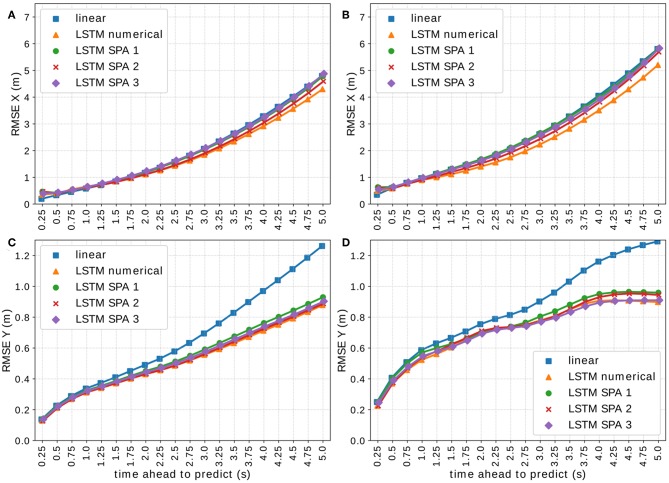
Visualization of the RMSE of all LSTM models on the complete *On-board* validation set *V*_1_ ⊂ *D*_1_ in *x*- **(A)** and *y*-direction **(C)** and on a subset of situations with at least 3 other vehicles present and distance between the target and ego vehicle lower than 20 m and between target and closest other vehicle lower than 10 m, again in *x*- **(B)** and *y*-direction **(D)**.

In this section, we describe the training process and parameters of all our models and give a detailed analysis and evaluation of the results achieved. The LSTM models are implemented in Tensorflow (Abadi et al., 2016) whereas the NEF models and the mixture-of-experts online learning model are implemented using the Nengo software suite (Bekolay et al., 2014). We use the root-mean-square error (RMSE) as our main metric for evaluation purposes. In contrast to earlier work, we inspect the RMSE for lateral and longitudinal directions separately to give more detailed insights into the models' behavior. Calculating the RMSE of the Euclidean distance would absorb the influence of the lateral RMSE since it is an order of magnitude smaller than the longitudinal RMSE, while we consider both directions to be at least equally important. The lateral RMSE is even more informative regarding the models' performance on, for instance, lane change maneuvers. Note however, that this means that the *y*-axes in Figures 5, 6, **8**–**11** show a different order of magnitude for lateral (RMSE X) and longitudinal (RMSE Y) direction. Finally, we investigate where the models shows their best performance looking for correlations between prediction accuracy and specific driving situations.

Table 1 summarizes the models evaluated in this section. The models LSTM SPA 1–3 as well as LSTM numerical employ the same network architecture as described in section 2.2.1 with sequential information as input data (using the different encoding schemes presented in section 2.3.4) and are analyzed in section 3.2.1. The models NEF SPA 1 and 2 employ the simpler, single-layer, feed-forward architecture as described in section 2.2.2 with a vector obtained as partial sum of vectors from the whole sequence used as input for the LSTM models (see section 3.1.2 for further details). Finally, mix online denotes the mixture-of-experts online learning model as described in section 2.2.3 using the predictions from some of the aforementioned offline models as input (see section 3.1.3 for further details). The models will be denoted in figure legends by their short name given in Table 1.

**Table 1:** Summary of the evaluated models regarding architecture, input data, encoding, and training.

**Short name**	**Input**	**Position encoding**	**Network architecture**	**Training**	**Number of units/Neurons**	**Data set**
Linear	Current position and velocity	–	Linear regression	–	–	Both
LSTM numerical	Sequence of positions	–	LSTM with one encoder/decoder cell each	Offline, backpropagation	150 units per cell	Both
LSTM SPA 1	Semantic vector sequence	Convolutive power	LSTM with one encoder/decoder cell each	Offline, backpropagation	150 units per cell	Both
LSTM SPA 2	Semantic vector sequence	Scalar multiplication	LSTM with one encoder/decoder cell each	Offline, backpropagation	150 units per cell	Both
LSTM SPA 3	Semantic vector sequence	Convolutive power incl. ego-vehicle	LSTM with one encoder/decoder cell each	Offline, backpropagation	150 units per cell	*On-board*
NEF numerical	Sequence of positions	–	NEF single-layer	Offline, least-squares	3,000 neurons	Both
NEF SPA 1	Semantic vector sum	Convolutive power incl. ego-vehicle	NEF single-layer	Offline, least-squares	3,000 neurons	*On-board*
NEF SPA 2	Semantic vector sum	Convolutive power	NEF single-layer	Offline, least-squares	3,000 neurons	*NGSIM*
Mix online	Predictions offline models	–	NEF single-layer	Online, delta-rule	3,000 neurons	Both

In section 2.3.4, we have described the different encoding schemes we will use to evaluate our models. We mentioned that the models employing the convolutive power to encode the input data are (i.e., LSTM SPA 1, 3 and NEF SPA 1 and 2) are the only ones having access to information about objects other than the target vehicle. Although these model therefore have access to more data than the other reference models, such as LSTM numerical, we are interested in evaluating the benefits of encoding the interconnections between vehicles implicitly in the input data using our semantic vector encoding instead of introducing a more complex network architecture. Therefore, we focus on the same network architecture for all encoding schemes in this paper and leave a comparison with more sophisticated network architectures, for instance, ones combining LSTM with social pooling layers as in Deo and Trivedi (2018a) or Alahi et al. (2016) for future work.

### 3.1. Model Training

#### 3.1.1. LSTM Networks

We trained several instantiations of our LSTM-network architecture as described in section 2.2.1 on the *On-board* data set *D*_1_ in advance to find an optimal set of parameters. We varied the number of layers, the number of hidden dimensions and the number of epochs for the models to be trained. We found, that increasing the number of layers does not improve the models' performance on the validation data, even when training longer using more epochs. On the contrary, models with more layers needed more training time to achieve a performance on the validation data comparable to the networks with less layers. Thus, a LSTM model with one encoder and decoder cell each is not only the simplest network architecture but also the best in terms of accuracy as well as time needed for training.

For this architecture, we found that the network performs best with 150 dimensions in the encoder and decoder cell each. Furthermore, we employed early stopping, that is, we trained our models for 10 epochs as we found that the models' performance stagnate on both, training and validation data sets, when training for up until a total 20 epochs. Figure 5 visualizes this result by showing the development of the RMSE of the LSTM SPA 1 model during the training process for the training set *T*_1_ (Figures 5A,C) and validation set *V*_1_ (Figures 5B,D) of the *On-board* data set *D*_1_. On the *y*-axis of each sub-figure, we have the RMSE while the *x*-axis from left to right depicts the result after each epoch during the training process. Each colored line illustrates the RMSE of the model for one particular prediction time step while all points with the same value on the *x*-axis depict the model's performance after the respective epoch during the training process.

Using the aforementioned network architecture and hyperparameter set, we train one model instantiation for each encoding scheme mentioned in section 2.3.4, whereas only the input dimensionality of the encoder cell changes when varying the representation of the input data. Importantly, we focus on positional information as the only input for our LSTM models in this work for reasons of consistency to make all models as comparable as possible. Hence, we neglect for example the history of the target (or ego-) vehicle's velocity or acceleration as input here. Between the two data sets, the only difference between models is the auxiliary data, that is used as additional input to the LSTM decoder cell at each time step. For both data sets, we use the instantaneous velocity of the target vehicle to aid the model predicting the future trajectory at every time step. As there is no ego-vehicle present, we use no further auxiliary data for the *NGSIM* data set *D*_2_. For the *On-board* data set *D*_1_, we use the ego-vehicle's predicted acceleration and the estimated curvature of the ego-vehicle's current lane. Although this is future information, we argue that it is solely about the ego-vehicle, which we expect to be available at the time the prediction is to happen. We assume, that an automated vehicle, in order to safely navigate, will have an estimation of the future lane curvature as well as the acceleration values of its own planned trajectory.

#### 3.1.2. NEF Networks

For our NEF networks, the main parameters influencing the models' performance are the number of neurons in the learning population (i.e., the hidden layer in terms of traditional neural networks), and the neuron model. For simplicity, we use Nengo's rate-variant of the leaky-integrate-and-fire (LIF) neuron model. From the NEF-theory (Eliasmith and Anderson, 2003) we know that increasing the number of neurons in a population yields a more accurate representation of the data encoded in the population's activity. Thus, we expect more accurate predictions when increasing the number of neurons. In our experiments, we found that a number of 3,000 spiking neurons is sufficient and further increasing the number of neurons does not improve the model's prediction accuracy. The neural weights are calculated using Nengo's default least-squares-optimization method with the exception, that we calculate the weights over smaller subsets of the input data for computational reasons.

We train two different variants of our simpler NEF-models using numerical input (NEF SPA numerical) as well as the SPA-power-with-ego (NEF SPA 1) and SPA-power encoding (NEF SPA 2) for the *On-board* and the *NGSIM* data set, respectively. Here, we adapt the input data such that for the model NEF numerical, we use the vector (*x*_*t*_0__, …, *x*_*t*_*N*__, *y*_*t*_0__, …, *y*_*t*_*N*__, *v*) as input with *v* denoting the instantaneous velocity of the target vehicle. For the NEF SPA 1 and 2 models, instead of flattening the whole sequence of vectors into a giant single vector, we created a single semantic vector by summing the first, middle, and last element of the original vector sequences

(10) $$\begin{array}{r} \hat{\text{S}}={\text{S}}_{t_{0}}⊕{\text{S}}_{t_{{}^{N}/{}_{2}}}⊕{\text{S}}_{t_{N}}=\left({ŝ}_{0},\ldots ,{ŝ}_{D-1}\right). \end{array}$$

We only sum up these vectors instead of the whole sequence ${\left({\text{S}}_{t}\right)}_{t_{0}}^{t_{N}}$ to avoid the accumulation of noise in the vector representation. Note that thereby the NEF model using the SPA-power representation does not use the full trajectory history but only selected time steps, namely those visualized in Figure 2. To make these simpler models as comparable as possible to the LSTM models in terms of information available to the network, we add the instantaneously velocity *v* of the target vehicle as an additional element to the input, which yields (ŝ_0_, …, ŝ_*D*−1_, *v*) as input of our model, since there is no intermediate embedding vector here where it could be included.

#### 3.1.3. Mixture-of-Experts Online Learning

**Figure 7:**
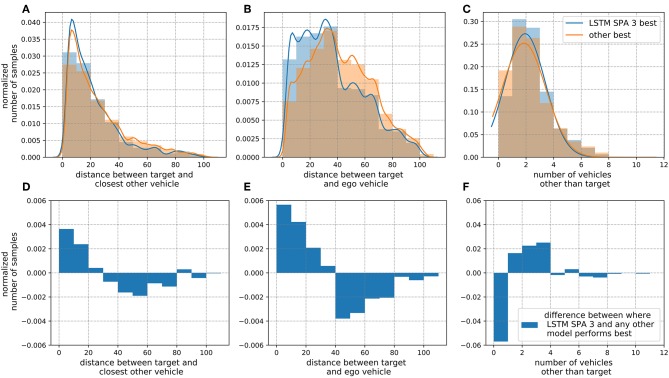
Metric evaluation specifying situations where the LSTM SPA 3 model outperforms all other approaches regarding the RMSE in *y*-direction on the *On-board* data set *D*_1_. In the upper row **(A–C)**, blue bars illustrate samples where LSTM SPA 3 performs better than all other models while the orange bars depict samples where any other model performs best. Panel **(A)** illustrates the distance between the target vehicle and the closest other vehicle, **(B)** illustrates the distance between the target and the ego-vehicle and **(C)** shows the number of vehicles other than the target. The lower row **(D–F)** illustrates the difference between the blue and orange bars in the corresponding upper panel.

There are two different possible variants to our mixture-of-experts online learning model. One issue of such a learning system is that the actual position information of the target vehicle **v**_*observed,t*_ and thus the error ϵ_*t*_ in Equation (9) is not available at the time the model makes its predictions, since it is future data. In this paper, we show a first prototype that, for simplicity, ignores this delay issue and assumes that position information of the target vehicle **v**_*observed,t*_ actually is available at prediction time. In the future, we aim to investigate an online learning system that updates its weights **W**_*p,t*_ once the error signal ϵ_*t*_ gradually becomes available. However, the architecture of the model itself remains the same. The only difference to the prototype shown here is the time when Equation (9) is applied to update the neural weights. For the context-sensitive mixture-of-experts model, we use information about the current driving situation as identified in section 3.2.1 and Figure 7 as context **c** for the learning system. For the *NGSIM* data set, we use the distance between the target-vehicle and the closest other vehicle as well as the number of surrounding relevant vehicles as context information. Relevant means that those vehicles that are included in the SPA-power representation are counted (see section 2.3.4). For the *On-board* data set, the distance between the target and the ego-vehicle is additionally included in the context.

In this work, we employ the pre-trained LSTM models using numerical inputs (i.e., LSTM numerical), the best-performing SPA-power encoding scheme for each data set (i.e., LSTM SPA 3 for the *On-board* data set and LSTM SPA 1 for the *NGSIM* data set), and a simple linear prediction as input experts for our online learning prototype. For training the model, we simulate online deployment by presenting the offline models' predictions on the validation subsets to the system. Thereby, the individual experts perform their prediction on previously unseen data samples. We conduct individual simulation runs for both data sets.

### 3.2. Evaluation

In this section, we evaluate the performance of our models and conduct a thorough analysis of the results achieved. For all evaluations in this section, we refer to the longitudinal and lateral direction as *x*- and *y*-direction, respectively.

#### 3.2.1. LSTM Models

Figure 6 visualizes the RMSE of all LSTM-based models on the validation-set *V*_1_ ⊂ *D*_1_ of the *On-board* data set using 512-dimensional vectors. Figures 6A,C show the performance on the complete validation-set in *x*- and *y*-direction, respectively, whereas Figures 6B,D depict only situations with at least 3 other vehicles present, the distance between the target and the ego-vehicle being lower than 20 m and the distance between the target and the closest other vehicle being <10 m, again for *x*- and *y*-direction, respectively. We observe that all approaches yield comparable results with notable differences in certain situations. Although the SPA-power encoding schemes (LSTM SPA 1 and 3) tend to perform worst in *x*-direction, we observe that they perform better in *y*-direction in crowded situations with closely driving vehicles with LSTM SPA 3 ranking best along LSTM numerical.

To further investigate this result, we evaluated certain metrics, chosen to characterize crowded and potentially dangerous situations, for items in the validation set, where the LSTM SPA 3 model outperforms all other approaches with respect to the RMSE in *y*-direction (see Figure 7). We observe that the number of samples, where the distance between the target and the ego vehicle and/or the closest other object being small is significantly higher when the LSTM SPA 3 model outperforms all other approaches. For samples where the LSTM SPA 3 model performs best, the number of samples with a distance <20 m between the target- and ego-vehicle is 50.5 % higher compared to samples where any of the other models performs best. For distances <20 m between the target vehicle and the closest other vehicle, the number of samples is still 11.4 % higher when the LSTM SPA 3 model performs best. Finally, the number of situations with at least 3 other vehicles present is also 7.8 % higher compared to samples where any other model performs best. Thus, we consider these characteristics suitable candidates to serve as context variables on which our online-learning mixture-of-experts system could base its weighting decision on. However, we aim to investigate more sophisticated options, such as clustering methods in future work to uncover other, potentially more meaningful features compared to the ones shown in this paper, distinguishing between situations where LSTM SPA 3 performs best compared to another model showing the best performance.

**Figure 8:**
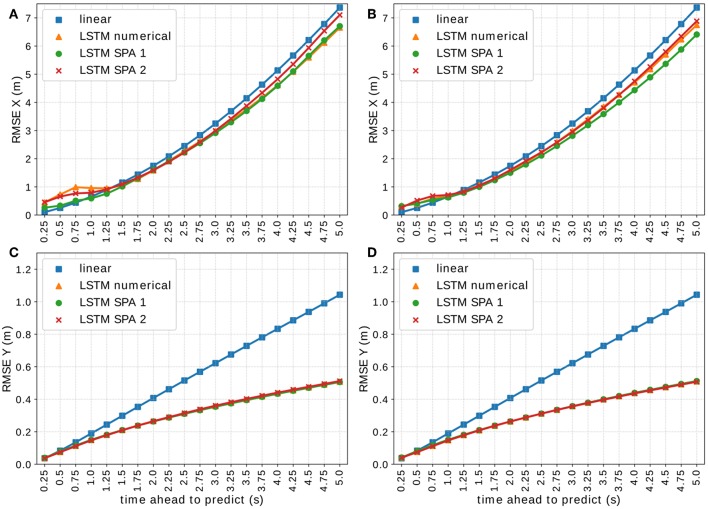
Visualization of the RMSE of all LSTM models on the *NGSIM* validation set *V*_2_ ⊂ *D*_2_ using vectors of dimension 512 for the LSTM SPA 1 and 2 models in *x*- **(A)** and *y*-direction **(C)** and using vectors of dimension 1,024 for the LSTM SPA 1 and 2 models in *x*- **(B)** and *y*-direction **(D)**.

Figure 8 visualizes the RMSE of all LSTM-based models on the validation-set *V*_2_ ⊂ *D*_2_ of the *NGSIM* data set for 512-dimensional vectors (Figures 8A,C) and for 1,024-dimensional vectors (Figures 8B,D). We observe, that all LSTM models achieve a very similar performance (almost identical in *y*-direction) with LSTM SPA 1 achieving the best performance in *x*-direction being on par with the numerical encoding for 512-dimensional vectors. For 1,024-dimensional vectors, LSTM SPA 1 even slightly outperforms all other approaches in *x*-direction, whereas we do not observe significant improvements in *y*-direction.

#### 3.2.2. NEF Networks

**Figure 9:**
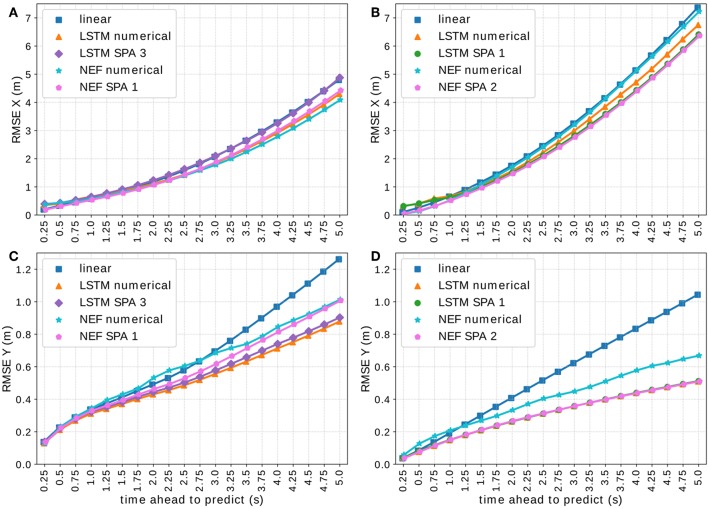
Visualization of the RMSE in *x*- **(A,B)** and *y*-direction **(C,D)** of the NEF SPA 1 model on the *On-board* validation set *V*_1_ ⊂ *D*_1_ using 512-dimensional vectors for the SPA-power vectors **(A,C)** and the NEF SPA 2 model on the *NGSIM* data set *D*_2_ using 1,024-dimensional vectors for the SPA-power vectors **(B,D)**.

Figure 9 visualizes the RMSE of our NEF-network models on both data sets. The NEF-network using the SPA-power encoding schemes processes 512-dimensional for the *On-board* (NEF SPA 1) and 1,024-dimensional vectors for the *NGSIM* data set (NEF SPA 2). For reference, we included the performance of the most relevant LSTM models, namely LSTM SPA 1 and 3 for the *NGSIM* and *On-board* data set, respectively as well as LSTM numerical, in Figure 9 as well. We observe that, despite a simpler network architecture and learning algorithm, the NEF-networks achieve a performance comparable to the more sophisticated LSTM models on both data sets. For the *NGSIM* data set, the NEF SPA 1 model performs on par with its LSTM model counterpart LSTM SPA 3. In this case, the NEF-model is not only simpler, but also has access to less information as its input data is a sum of a subset of the input sequence used for the corresponding LSTM-model.

#### 3.2.3. Mixture-of-Experts Online Learning

**Figure 10:**
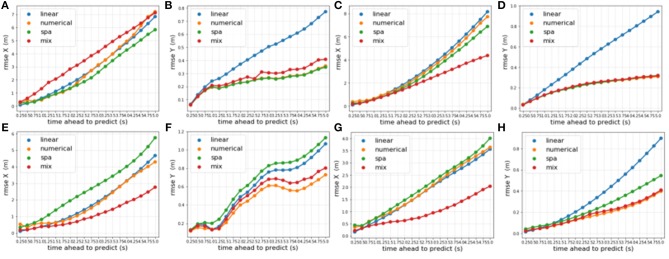
Visualization of the RMSE of the context-sensitive mixture-of-experts online learning system on selected data-slices from the validation sets. The upper row shows the RMSE in *x*-direction **(A–D)**, while the lower row shows the RMSE in *y*-direction **(E–H)**. Panels **(A,E)** show the RMSE on the *On-board* data set at the start of training process while **(B,F)** show the RMSE performance on the first 70 vehicles. Similarly, panel **(C,G)** show the RMSE on the *NGSIM* data set at the start of the training process while **(D,H)** show the RMSE on the first 92 vehicles.

Figure 10 shows the RMSE on selected slices of the validation-sets achieved by our context-sensitive mixture-of-experts online learning prototype, which assumes the error signal is available at the time the prediction needs to happen in comparison to the offline models. The four left plots (Figures 10A,B,E,F) show two data slices of the validation set *D*_1_ of the *On-board* data set: Figures 10A,E show the RMSE at the start of training process while Figures 10B,F show the RMSE performance on the first 70 vehicles. Similarly, the four right plots (Figures 10C,D,G,H) show two data slices of the validation set *D*_2_ of the *NGSIM* data set: Figures 10C,G show the RMSE at the start of the training process while Figures 10D,H show the RMSE on the first 92 vehicles. From Figures 10A,E,C,G we observe, that the model needs some time for adapting its weights yielding a RMSE performance worse than the individual experts for both data sets. However, the model's performance improves quickly and clearly outperforms all individual experts in *x*-direction while achieving RMSEs as low as the best individual experts in *y*-direction after a comparably low number of vehicles presented to the system. Figures 10B,F illustrate this result for the *On-board* data set, while Figures 10D,H show comparable results achieved by the mixture model on the *NGSIM* data set.

**Figure 11:**
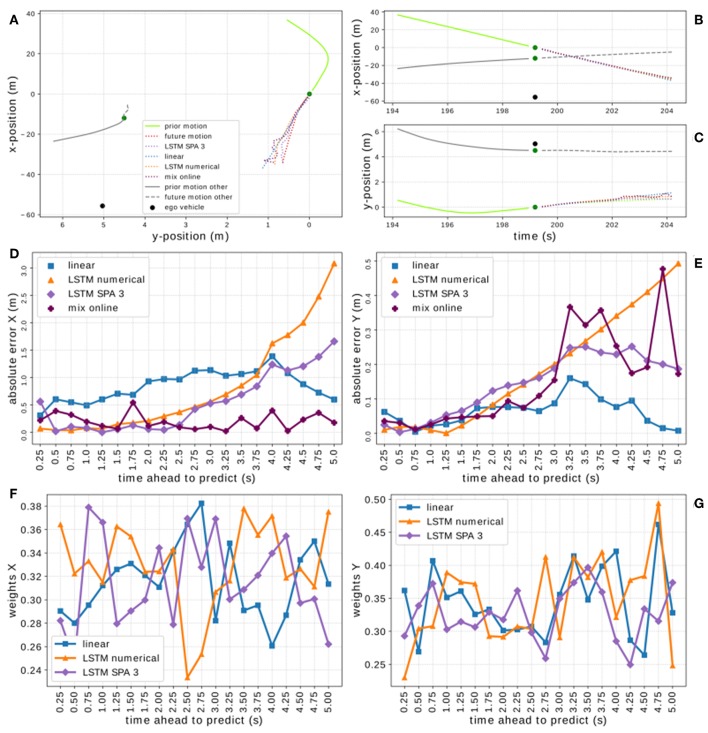
Visualization of the context-sensitive mixture-of-experts online learning system on one particular driving situation from the *On-board* data set. Panels **(A–C)** depict the driving situation with the vehicles' true trajectories as well as the trajectory predictions given by the offline models and the mixture-of-experts online learning prototype. Panels **(D,E)** show the absolute error of all prediction models on that data-sample. Panels **(F,G)** visualize, how the mixture model weights the individual experts for every prediction time step in this particular driving situation.

To get a better idea of how our model weights the individual predictors, we inspect one example driving situation. Figure 11 visualizes the performance of our mixture-of-experts online learning prototype on one particular example of the *On-board* data set. We use a situation not directly after the start of the training process, i.e., the mixture model was already exposed to some vehicles and thus was able to consolidate its weights. Figures 11A–C show the driving situation with the vehicles' true trajectories as well as the trajectory predictions given by the offline models and the mixture-of-experts online learning prototype. Figures 11D,E show the absolute error of all approaches while Figures 11F,G visualize, how the model weights the individual experts for every prediction time step in this particular driving situation. We observe that the overall trend of our model shows in this example as well. The mixture-of-experts prototype achieves significant improvements in the *x*-direction while achieving RMSEs comparably low as the best individual expert in *y*-direction (Figures 11D,E). Furthermore, Figures 11F,G show that the model weights the expert predictors independently at individual time steps and hence is able to pick the best possible predictor at each time step. However, we also observe, that the error of the mixture-of-experts model in the *y*-direction is higher than the best individual predictor and that the weighting, especially for later prediction steps, could be improved.

## 4. Discussion

For both data sets used in this paper, we observe that already the simple linear prediction models achieve solid accuracy, especially in longitudinal direction. This makes sense as both data sets almost exclusively contain highway driving situations, which in turn consist mainly of straight driving and rather rare lane-change maneuvers. For straight driving, linear prediction based on a constant velocity assumption is already a solid prediction approach, especially if all dynamic information (position, velocity etc.) are given relative to an already moving ego-vehicle like with the *On-board* data set *D*_1_. Table 2 summarizes the composition of both data sets.

**Table 2:** Composition of both data sets regarding straight driving and samples containing a lane change performed by the target vehicle.

**Data set**	**Straight driving**	**Total target vehicle lane changes**	**Past target vehicle lane changes**	**Future target vehicle lane changes**
*On-board*	86.1 %	13.9 %	7 %	8.2 %
*NGSIM*	95.1 %	4.9 %	2.7 %	2.6 %

For the *On-board* data set, in 86.1 % of all data samples the target vehicle does not perform a lane, i.e., only 13.8 % of all data samples contain a lane change performed by the target vehicle. We further distinguish between lane changes performed during the trajectory history, i.e., the past 5 s before the current time step (labeled as *past* in Table 2) and lane changes that are performed in the future, i.e., the future 5 s from the current time step (labeled as *future* in Table 2). For the *NGSIM*, the percentage of samples without a target vehicle lane change is 95.1 % while only 4.9 % of the samples contain a lane change performed by the target vehicle at all. The amount of samples containing a future lane change performed by the target vehicle is only 2.6 % of all samples in the *NGSIM* data set.

For the offline models, simple feed-forward NEF models and more sophisticated LSTM models alike, we observe that most improvements over the linear model are achieved in *y*-direction. That makes sense as linear prediction is unable to account for lane-changes or driving curves, which are mainly characterized by non-linear changes in lateral direction. We found that the LSTM models based on our SPA-power representation (LSTM SPA 1 and 3) achieve promising results on both data sets. However, for the *On-board* data set, this encoding scheme achieves its best result in crowded and potentially dangerous driving situations, without clearly outperforming the other approaches on the whole data set (see section 3.2.1 and Figure 6). Given these finding, we investigated situations, where the LSTM SPA 3 model does outperform all other approaches in *y*-direction and thereby came up with metrics characterizing such crowded situations (see Figure 7). This result did not hold that clearly on the *NGSIM* data set *D*_2_, since the LSTM models achieve an almost identical performance in *y*-direction on this data set.

Nevertheless, we used the identified characteristics as context information for our first prototype of a mixture-of-experts online learning system based on simple delta-rule learning. For simplicity, the prototypical model shown here ignores the fact that measurements of the actual trajectory and thus the error signal for the learning system is future data, i.e., only available with a timing delay, and applies Equation (8) instantaneously. We tested and evaluated this prototype on both data sets achieving comparable results. We found that already shortly after initialization, the online learning system is able to adapt its weights to significantly improve its performance over the individual expert systems. Interestingly, the mixture-of-experts model achieves the most improvements over the individual experts in the *x*-direction although the characteristics used as context were derived from analyzing the LSTM models' performance in the *y*-direction. We assume that this is due to the fact, that the individual LSTM experts already show a closer-to-optimal performance in the *y*-direction with less room for improvements. Furthermore, the sample situation shown in Figure 11 exemplifies another potential problem of the current model in the *y*-direction: with a distance of 12.8 m between the target and the closest other vehicle, a distance of 55.8 m between the target and the ego-vehicle and only one other vehicle present, this is not a typical situation for the LSTM SPA 3 model to perform best in the *y*-direction (cf. Figure 7) and thus this expert might not be weighted strongly enough by the model. However, these effects demand for further and more detailed investigation.

Another interesting result of our experiments is the fact, that the simple, feed-forward NEF networks show results comparable to the more sophisticated LSTM models. For those simple models, the SPA-power representation (NEF SPA 1 and 2) shows promising results comparable to the NEF numerical model on the *On-board* data set and clearly outperforming it on the *NGSIM* data set (Figure 9). Although the NEF models do not clearly outperform the LSTM models (which would be surprising), it is quite remarkable that they achieve results comparable to the more sophisticated models with a simpler network architecture, training procedure and, partly, less information. These results make those simple models using our proposed vector-representation as well as a numerical encoding scheme (possibly in combination with an online learning system like the one proposed in this paper) potential candidates to be deployed on dedicated neuromorphic hardware in mobile applications, as they can be efficiently implemented in a spiking neuron substrate. This could be an interesting, power-efficient approach in future automated vehicles.

### 4.1. Conclusion

In this paper, we showed a novel approach to encapsulate spatial information of multiple objects in a sequence of semantic pointers of fixed vector length. We used a LSTM sequence to sequence model as well as a simple feed-forward spiking neural network to predict future vehicle positions from this representation. For each of those models, we implemented at least one reference model using other encoding schemes to compare their performance to. Furthermore, we compared all our models to a simple linear prediction based on a constant velocity assumption. We evaluated our models on two different data sets, one recorded with on-board sensors from a driving vehicle and one publicly available trajectory data set recorded with an external camera observing a highway segment and conducted a thorough analysis. Finally, we used our pre-trained LSTM networks as basis for a mixture-of-experts online learning prototype and compared its performance to the individual expert systems. We consider our main contributions the proposed representation of spatial information for multiple objects in semantic vectors of fixed length using the convolutive power, the rigorous and detailed analysis of several simple and more advanced models, and the prototype of our online learning system.

### 4.2. Future Work

Although the results presented in this paper show promise, there are several directions for future work. Regarding our LSTM models, we aim to investigate if increasing the vector dimension further leads to improved model performance on the *On-board* data set, as the results on the *NGSIM* suggest that there is potential for improvements (see Figures 8A,B). Furthermore, our preliminary hyperparameter experiments suggest, that there is potential for improvements by incorporating the history of the target and/or ego-vehicle's velocity and/or acceleration. Therefore, we could investigate possibilities of how to encode such information in a semantic vector substrate. Another interesting option for the offline models is to investigate if a reduced, more balanced data set could improve the models accuracy or at least speed up the training process. As mentioned in section 4 and Table 2, both data sets are slightly unbalanced as they are dominated by straight driving, which is most common in highway situations. One possibility could be to use the current data sets and focus the training procedure on “interesting scenes,” i.e., situations where for example a lane change is happening by for instance looking for data samples with significant differences in the lateral positions. Another option is to improve our current models to predict a probability distribution of the future positions instead of point predictions of raw position values to take uncertainties into account. Finally, we could also compare our current models to other state-of-the-art models, which combine LSTM and social pooling layers, which we did not include in the work at hand.

Regarding our mixture-of-experts online learning prototype, we have shown a simplified version ignoring the fact that the error signal is future data and thus can not be used instantaneously, but rather becomes gradually available over time. Although the network architecture and learning approach would remain unchanged, the timing when the weights' update happens needs to be implemented and investigated if and how this affects the models performance. However, the results achieved in this paper serve as an upper bound for the performance to be expected from models that have to deal with delayed error signals, that is, that the target vehicle's true motion is future data and thus not available at prediction time, The issue of delayed error signals was mentioned but, for simplicity, not addressed in this work. However, assuming that a model overpredicting the near future most likely will also overpredict for later time steps, we could also experiment with model variants that update the weights for later prediction steps based on the error signal for earlier prediction steps before the error signal actually becomes available. Another direction could be to investigate if and how different context information affect the model's performance.

Since advanced driver assistance systems and, more generally, automated driving are mobile applications with tight energy restrictions, we finally aim to investigate if and how our current implementation could be deployed on dedicated, energy-efficient neuromorphic hardware for mobile, in-vehicle applications.

## Author Contributions

FM has designed and implemented all the model variants in Tensorflow and Nengo, designed and performed the experiments, pre-processed data, evaluated results, and wrote the manuscript. PB has designed the numerical LSTM models in Tensorflow and assisted in data pre-processing, experiments and evaluation, and revised the manuscript. TS has designed the models in Nengo, assisted in data pre-processing, experiments and evaluation, and contributed in writing the manuscript. JC coordinated and supervised the research work, and revised the manuscript.

### Conflict of Interest

FM was employed by BMW AG. PB and TS were employed by Applied Brain Research Inc. The remaining author declares that the research was conducted in the absence of any commercial or financial relationships that could be construed as a potential conflict of interest.
